# Comparison of the Effectiveness of Different Antidepressants in Preventing Psychiatric Rehospitalization

**DOI:** 10.7759/cureus.76200

**Published:** 2024-12-22

**Authors:** Hannah Mei, Jackie Rowe, Amanda Sowinski, Daniel J Greer

**Affiliations:** 1 Ernest Mario School of Pharmacy, Rutgers University, Piscataway, USA

**Keywords:** antidepressive agents, depression, hospitalization, patient readmission, psychiatry

## Abstract

Objective: Patients with major depressive disorder (MDD) often face poor health outcomes. Additionally, patients with multiple hospitalizations tend to have worse predicted disease prognosis. Antidepressant medications remain a first-line treatment option for MDD, but data evaluating the effects of different antidepressants on psychiatric readmission rates is lacking. The objective of this study was to assess readmission rates based on antidepressant selection upon discharge from an inpatient psychiatric hospitalization.

Methods: This was a single-center, retrospective chart review evaluating psychiatric readmission rates of adults with MDD discharged on antidepressant therapy. The primary outcome was to compare 30-day psychiatric rehospitalization rates based on antidepressant treatment and secondary outcomes included six-month and one-year psychiatric rehospitalization based on antidepressant treatment. Odds ratios (OR), 95% confidence intervals (CI), and p-values were calculated to determine the odds of readmission in patients who were discharged on each antidepressant compared to patients who were discharged on an antidepressant other than that particular antidepressant.

Results: Patients who were discharged on sertraline were four times less likely to be readmitted within 30 days compared to the combined readmission rate of other antidepressants or multiple antidepressants (OR: 0.228, 95%CI: 0.053-0.978, p<0.05). Meanwhile, patients discharged on any combination of multiple antidepressants were more likely to be readmitted than patients discharged on only one antidepressant (OR: 4.517, 95%CI: 1.581-12.908, p<0.05). The antidepressant medications included in the study were bupropion, duloxetine, escitalopram, fluoxetine, mirtazapine, paroxetine, sertraline, trazodone, and venlafaxine.

Conclusion: Careful consideration should be taken when choosing antidepressant therapy for inpatient psychiatric patients as this may impact relapse of symptoms and readmission rates. Further research is needed to evaluate other factors that may influence psychiatric readmissions for MDD.

## Introduction

Major depressive disorder (MDD) is a common psychiatric condition that can significantly impact patients’ health and quality of life and increase financial burden. The estimated prevalence of MDD among adults in the United States (US) is 8.3% [1]. MDD is diagnosed based on criteria that include symptoms such as a depressed mood or loss of interest in activities, along with other signs like changes in weight, insomnia or excessive sleep, psychomotor agitation or slowing, fatigue, feelings of guilt, reduced concentration, and thoughts of suicide. These symptoms must persist for at least two weeks and cause significant distress or impairment in daily functioning [2]. Medications along with psychotherapy are recommended treatments for depression. Various classes of antidepressant medications are available such as selective serotonin reuptake inhibitors (SSRIs), serotonin-norepinephrine reuptake inhibitors (SNRIs), atypical antidepressants, tricyclic antidepressants, and monoamine oxidase inhibitors. Although antidepressants are recommended as a first-line treatment for MDD, different antidepressants may lead to varying health outcomes. A meta-analysis of 522 trials compared efficacy and tolerability between antidepressant medications and found that all antidepressants were more effective than placebo; however, agomelatine (not available in the US), amitriptyline, escitalopram, mirtazapine, paroxetine, venlafaxine, and vortioxetine were more effective than other antidepressants [3]. Additionally, the study found that agomelatine, citalopram, escitalopram, fluoxetine, sertraline, and vortioxetine were more tolerable than other antidepressants.

If patients do not have full remission after treatment with one antidepressant, then antidepressant augmentation, also called antidepressant polypharmacy, may be trialed. Antidepressant augmentation is the pharmacologic strategy of the addition of a second antidepressant with two different mechanisms of action for synergistic and enhanced effects. A meta-analysis of 39 randomized controlled trials assessing the comparative efficacy and tolerability of combination antidepressant therapy found that combination treatment was superior to monotherapy based on a standardized mean difference in treatment outcomes [4]. This benefit of combination therapy was especially seen when a reuptake inhibitor (SSRI, SNRI, or tricyclic antidepressant) and an antagonist of presynaptic α2-autoreceptors (mianserin, mirtazapine, trazodone) were combined. Additionally, the study noted that dropout rates did not differ between treatment groups, which may indicate the tolerability of combinations [4].

Assessing which antidepressant medication or medications decrease readmission rates is important for the welfare of patients as well as for decreasing healthcare costs. Hospital readmissions for MDD may reflect an exacerbation or relapse and serve as a driver of healthcare costs [5]. Patients with multiple hospitalizations tend to have a worse predicted prognosis, as the recurrence rate of depression increases with each episode of depression [6]. A study by Lin et al. evaluated the effect of factors such as gender, marital status, and number of hospitalizations on time to readmission in patients with MDD [7]. The investigators only found that the number of previous hospitalizations was predictive of a shorter time to readmission. The Centers for Medicare & Medicaid Services publicly reports readmission rates and hospitals with high amounts of readmissions can lose a percentage of their reimbursement [8]. Also important to consider is the cost of the medication. Patients who are unable to afford medication after hospital discharge may be at an increased risk for readmission. A study by Vlahoitis et al. stated the average six-month healthcare cost is less for patients initiated on a generic antidepressant compared to those initiated on a brand-name antidepressant, costing $3660 and $4586, respectively [9]. The cost of an antidepressant varies greatly as seen in one review finding the annual cost ranges from $36-$1,603 depending on the antidepressant chosen [10]. Therefore, selecting an optimal treatment for MDD can help to reduce readmissions, improve health outcomes, and reduce healthcare costs.

The previously discussed factors of antidepressant choice, antidepressant augmentation, and cost of medications and care all influence readmission rates. The objective of this study is to evaluate 30-day, six-month, and one-year psychiatric readmission rates of patients discharged on different antidepressants for MDD at a large academic medical center.

## Materials and methods

This is a single-center, retrospective chart review of patients aged 18 years or older admitted with voluntary or involuntary status for a psychiatric exacerbation of MDD as diagnosed by the psychiatrists and discharged between August 1, 2020, and June 30, 2023, on at least one antidepressant medication. The location of the study is a single, large academic hospital, St. Joseph's University Medical Center, Paterson, New Jersey, US, with a 24-bed adult inpatient psychiatric unit that includes voluntary and involuntary admissions. The purpose of the study was to assess differences in readmission rates at a single institution among antidepressant medications prescribed at the time of discharge. The study was approved by the Rutgers University Office for Research (approval number: Pro2023001385).

Patient selection

Patients on any combination of more than one antidepressant at discharge were included as one group due to the potential for multiple combinations of medications. Patients were selected using convenience sampling. An admission was defined as admission to the inpatient psychiatric unit. Patients with a diagnosis of bipolar disorder, schizoaffective disorder, or schizophrenia in addition to MDD were excluded to limit the potential for misdiagnosis in the study population. Patients hospitalized for non-psychiatric reasons, prisoners, and pregnant patients were excluded. Only initial hospitalizations between August 1, 2020, and June 30, 2022, were assessed, and readmission data was evaluated through June 30, 2023, to account for one-year readmission rates following initial hospitalization.

Outcomes

The primary outcome assessed was 30-day psychiatric rehospitalization rates based on the antidepressant used at the time of initial discharge. Secondary outcomes were readmission within six months and one year based on the antidepressant used at the time of initial discharge. Readmissions that occurred more than 30 days after and within six months of initial hospitalization were considered a six-month rehospitalization, and those that occurred more than six months after and within one year of initial hospitalization were considered a one-year rehospitalization. Each readmission was only counted as a rehospitalization once (e.g., a 30-day rehospitalization was not also counted as a six-month rehospitalization despite it occurring within six months of initial hospitalization). The number of patients who were on the antidepressant being evaluated was considered the exposed group, while all other patients in the study were considered the non-exposed group. Suicidality was noted by Columbia-Suicide Severity Rating Scale (C-SSRS) screen version [11].

Data analysis

Odds ratios (OR), 95% confidence intervals (CI), and p-values were calculated using MedCalc (MedCalc Software Ltd, Ostend, Belgium) to determine the odds of readmission in patients who were discharged on each antidepressant compared to patients who were discharged on an antidepressant other than that particular antidepressant. The positive outcome used in the OR calculator was patients who were readmitted, and all other patients who were not readmitted were part of the negative outcome group. Descriptive statistics were calculated using IBM SPSS Statistics for Windows, Version 29.0 (Released 2023; IBM Corp., Armonk, New York, US). Statistical significance was defined as a p-value of less than 0.05.

## Results

A total of 319 patients were discharged on at least one antidepressant medication between August 1, 2020, and June 30, 2022. The mean age was 37 years and 53% were female (Table 1).

**Table 1:** Baseline characteristics of patients MDD: major depressive disorder; C-SSRS: Columbia-Suicide Severity Rating Scale

Category	Variable	Frequency (Percentage)
Sex	Female	169 (53.0%)
	Male	150 (47.0%)
MDD Diagnosis	Major depressive disorder, single episode, mild (F32.0)	1 (0.3%)
	Major depressive disorder, single episode, moderate (F32.1)	9 (2.8%)
	Major depressive disorder, single episode, severe without psychotic features (F32.2)	52 (16.3%)
	Major depressive disorder, single episode, severe with psychotic features (F32.3)	19 (6.0%)
	Major depressive disorder, single episode, unspecified (F32.9)	78 (24.5%)
	Depression, unspecified (F32.A)	6 (1.9%)
	Major depressive disorder, recurrent, moderate (F33.1)	9 (2.8%)
	Major depressive disorder, recurrent severe without psychotic features (F33.2)	103 (32.3%)
	Major depressive disorder, recurrent, severe with psychotic symptoms (F33.3)	28 (8.8%)
	Major depressive disorder, recurrent, in partial remission (F33.41)	1 (0.3%)
	Major depressive disorder, recurrent, unspecified (F33.9)	13 (4.1%)
Psychiatric Comorbidities	No	24 (7.5%)
	Yes	295 (92.5%)
Initial C-SSRS	Negative (answered “no” to all questions)	61 (19.1%)
	Positive (answered “yes” to ≥1 questions)	258 (80.9%)

The most common MDD diagnosis was MDD recurrent severe without psychotic features (32.3%). Of the study participants, 92.5% had at least one psychiatric comorbidity and over 80% of the patients answered “yes” to at least one question in the C-SSRS screen version upon admission, indicating some level of risk for suicide. Antidepressant agents, dose, duration of therapy, and length of stay can be found in Table 2. Doses reviewed in this study are expressed as milligrams (mg). 

**Table 2:** Antidepressant treatment doses and duration and length of stay

Antidepressant	Average Dose (mg)	Dose Range (mg)	Average duration of therapy (days)	Average length of stay (days)
Bupropion	190	150-300	5.53	5.80
Duloxetine	48.75	20-100	4.38	3.88
Escitalopram	13.17	5-20	4.18	5.14
Fluoxetine	24.35	10-60	3.48	4.87
Mirtazapine	17.93	7.5-45	4.70	6.74
Paroxetine	36.25	25-60	13.00	13.00
Sertraline	56.25	25-200	3.58	5.40
Trazodone	122.22	50-200	2.56	6.33
Venlafaxine	175	75-300	3.50	3.83
>1 antidepressant				4.17

All antidepressants fell within the normal dose range used for the treatment of MDD, with the exception of one patient who received 20 mg doses of duloxetine (minimum dose for MDD is 30 mg). While 20 patients had a six-month readmission for MDD, only seven patients had a 30-day readmission, and only nine had a one-year readmission. Information on antidepressants and number of readmissions is shown in Table 3.

**Table 3:** Number of readmissions associated with each antidepressant treatment

Antidepressant	Number of Patients	Number of Readmissions			
		30-day	6-month	1-year	Total, n (%)
Bupropion	15	2	1	0	3 (20.0%)
Duloxetine	8	0	1	0	1 (12.5%)
Escitalopram	153	4	10	2	16 (10.5%)
Fluoxetine	23	0	2	1	3 (13.0%)
Mirtazapine	23	0	2	1	3 (13.0%)
Paroxetine	4	0	0	0	0 (0.0%)
Sertraline	60	0	1	1	2 (3.3%)
Trazodone	9	0	0	1	1 (11.1%)
Venlafaxine	6	0	0	1	1 (16.7%)
>1 antidepressant	18	1	3	2	6 (33.3%)
Total	319	7	20	9	36 (11.3%)

Patients discharged on sertraline had statistically significant lower odds of readmission when compared to patients discharged on any other antidepressant or more than one antidepressant (OR: 0.228, 95%CI: 0.053-0.978, p<0.05) (Table 4). Patients discharged on more than one antidepressant in any combination had higher rates of readmission when compared to patients discharged on antidepressant monotherapy (OR: 4.517, 95%CI: 1.581-12.908, p < 0.05). Of note, fluoxetine, mirtazapine, paroxetine, trazodone, and venlafaxine did not have any 30-day readmissions, though the sample size was too small to find a significant difference in the OR calculation. 

**Table 4:** Odds ratios of readmissions based on antidepressant medication *Statistical significance was defined as a p-value of less than 0.05

Antidepressant	OR (95% CI)	p-value
Bupropion	2.053 (0.551-7.653)	0.284
Duloxetine	1.127(0.135-9.429)	0.913
Escitalopram	0.853(0.424-1.713)	0.654
Fluoxetine	1.196 (0.337-4.241)	0.782
Mirtazapine	1.196 (0.337-4.241)	0.782
Paroxetine	0.851 (0.045-16.128)	0.914
Sertraline	0.228 (0.053-0.978)	0.047*
Trazodone	0.982 (0.119-8.088)	0.987
Venlafaxine	1.589 (0.180-13.992)	0.677
>1 antidepressant	4.517 (1.581-12.908)	0.005*

## Discussion

In this study, patients discharged on sertraline were less likely to be readmitted within 30 days compared to patients who were discharged on other antidepressants. These findings are consistent with published data. A study conducted by Warnke et al. in Germany analyzed health insurance claims data of patients with unipolar depression and compared the times to readmission among individual antidepressants and found that sertraline was the only antidepressant shown to reduce readmission risk, as it was associated with a 37% increase in time to readmission and a 40% reduction in the probability of hospitalization in a given week [12]. A retrospective cohort study conducted by Zhu et al. aimed to predict individual psychiatric readmission within 30, 60, 90, 180, and 365 days of an initial psychiatric hospitalization for MDD and reported that the use of fluoxetine during index admissions showed a strong association with the risk of psychiatric readmission [5]. Lastly, in a cohort study by Tiihonen et al., which evaluated the effectiveness of different pharmacological treatments on relapse prevention in patients who had been hospitalized for unipolar depression, while antidepressants were not associated with a reduced risk of readmission, further analysis of each antidepressant did reveal that some antidepressants were associated with a lower risk of readmission [13]. The authors included an incident cohort to analyze a subpopulation of new patients in an effort to avoid survival bias and found that out of all the antidepressants, patients who were prescribed amitriptyline or sertraline were shown to be associated with the lowest risk of readmission. A limitation of all three studies is that each study was conducted in a different country, where prescribing practices for antidepressants may differ from those in the US. Additionally, these studies did not include all antidepressants that are commonly prescribed in the US for MDD. Furthermore, the patient demographics in each study differ from the patient population in the US, causing difficulty in extrapolating the results of these studies in the US patient population. Further research is needed to evaluate the potential effects of sertraline on reducing readmission for MDD.

Patients in the current study discharged on antidepressant combination therapy had higher odds of readmission when compared to patients who were discharged on antidepressant monotherapy. Antidepressant combinations increase the risk of drug-drug interactions, which may decrease the treatment’s efficacy or increase the prevalence of adverse effects. The American Psychiatric Association’s Practice Guideline only recommends antidepressant polypharmacy for patients with treatment-resistant depression who may benefit from the synergistic effect of using antidepressants with different mechanisms of action [14]. Monotherapy is typically trialed first but psychiatrists may consider augmenting the first antidepressant with a second antidepressant with a different mechanism of action. This strategy is sometimes trialed if the patient experiences partial benefit with relatively mild side effects from the first antidepressant [15]. One potential explanation for the finding in the current study is that these patients were discharged on more than one antidepressant because they had a more severe case of MDD, so their chances of readmission may have been higher than patients who were discharged on only one antidepressant. Another potential explanation is that these patients were prescribed more than one antidepressant because they did not have an optimal response to the first antidepressant, and it could be possible that these patients were still not responding well to the medications, which would put them at a higher risk of readmission.

The economic burden of MDD among adults continues to increase in the US. Greenberg, et al. reported a 37.9% increase from $236.6 billion in 2010 to $326.2 billion in 2018 (2020 US dollars), and further increased to $333.7 billion in 2019, which equates to $16,854 per adult [16]. A cost breakdown of all the direct and indirect costs associated with MDD in the US by Greenberg reveals that healthcare costs make up the largest percentage of the total economic burden in 2019, accounting for $127.3 billion [17]. Healthcare costs include prescription drugs, outpatient services, and hospitalizations. Thus, identifying an antidepressant with lower readmission rates may reduce direct healthcare costs, and improve healthcare outcomes that could further reduce indirect costs associated with MDD.

The strengths of the study included the defined inclusion criteria, which eliminated patients using antidepressants for other indications. Additionally, the sample size included 319 patients discharged on antidepressant medications. The multiple time frames for readmission of 30 days, six months, and one year allows a more comprehensive understanding of readmissions. The implications of this study are to support the design of larger, multicenter studies aimed at identifying the factors that contribute to psychiatric readmissions.

There were several limitations of note. The typical time to response after initiation of an antidepressant is at least four weeks. Therefore, some readmissions that occurred within 30 days may have been due to the patient’s condition not responding to treatment since the medication has not produced its full effects yet. The availability of information also could have influenced the results of this study. Since all of the data included was collected from the electronic health records, this study was unable to account for any information regarding the patient prior to admission or after discharge, such as patients’ medication adherence and the dose, duration, and type of antidepressants that patients were on outside this hospital. Since the hospital's electronic health record system only records admissions to this hospital, any admissions to other hospitals during this study period were unaccounted for in the analysis. This study did not include information on patients’ insurance and employment status, which could have influenced how likely patients were readmitted to the hospital. For example, patients who did not have insurance and relied on charity care through the hospital could have been readmitted more often since they could only obtain treatment and medications while they were admitted to the hospital. These patients may not have necessarily been admitted due to an MDD exacerbation even though the admission may have been documented as one. Additionally, this was a single-center trial with a relatively small sample size and a larger analysis would be beneficial in the future. For example, there were no 30-day readmissions for patients discharged on fluoxetine, mirtazapine, paroxetine, trazodone, and venlafaxine, but the sample size was too small for these agents to find a significant difference in OR. Finally, sertraline was the second most commonly prescribed antidepressant, and therefore results may differ at institutions where other agents are prescribed more frequently.

A high-level review and further analysis would be helpful to determine the full implications of antidepressant selection. Antidepressant selection is based on many factors such as renal function, hepatic function, previous medication trials, family response to other antidepressants, and concomitant illnesses. Future analysis that addresses each of the many variables would be beneficial.

## Conclusions

Of the antidepressants assessed in this study, only sertraline was shown to be associated with lower odds of readmission in comparison to other antidepressants. Meanwhile, patients who were discharged on multiple antidepressants had higher odds of readmission than patients discharged on one antidepressant. Further research is warranted to assess other factors that may influence psychiatric readmissions in patients with MDD.

## References

[1] (2024). National Institute of Mental Health: Major depression. https://www.nimh.nih.gov/health/statistics/major-depression.

[2] (2013). Depressive disorders. Diagnostic and Statistical Manual of Mental Disorders (5th edition).

[3] Cipriani A, Furukawa TA, Salanti G (2018). Comparative efficacy and acceptability of 21 antidepressant drugs for the acute treatment of adults with major depressive disorder: a systematic review and network meta-analysis. Lancet.

[4] Henssler J, Alexander D, Schwarzer G, Bschor T, Baethge C (2022). Combining antidepressants vs antidepressant monotherapy for treatment of patients with acute depression: a systematic review and meta-analysis. JAMA Psychiatry.

[5] Zhu T, Jiang J, Hu Y, Zhang W (2022). Individualized prediction of psychiatric readmissions for patients with major depressive disorder: a 10-year retrospective cohort study. Transl Psychiatry.

[6] Bains N, Abdijadid S (2023). Major depressive disorder. StatPearls [Internet].

[7] Lin CH, Chen YS, Lin CH, Lin KS (2007). Factors affecting time to rehospitalization for patients with major depressive disorder. Psychiatry Clin Neurosci.

[8] Bradley EH, Curry L, Horwitz LI (2013). Hospital strategies associated with 30-day readmission rates for patients with heart failure. Circ Cardiovasc Qual Outcomes.

[9] Vlahiotis A, Devine ST, Eichholz J, Kautzner A (2011). Discontinuation rates and health care costs in adult patients starting generic versus brand SSRI or SNRI antidepressants in commercial health plans. J Manag Care Pharm.

[10] (2020). Appendix 1: cost comparison. Pharmacoeconomic Review Report: Vortioxetine Hydrobromide (Trintellix): (Lundbeck Canada Inc.): Indication: The treatment of Major Depressive Disorder in adults.

[11] (2008). Columbia-Suicide Severity Rating Scale.

[12] Warnke I, Nordt C, Moock J, Kawohl W, Rössler W (2014). Antidepressants: relationship to the time to psychiatric readmission and probability of being in hospital in depressive patients. Front Public Health.

[13] Tiihonen J, Tanskanen A, Hoti F (2017). Pharmacological treatments and risk of readmission to hospital for unipolar depression in Finland: a nationwide cohort study. Lancet Psychiatry.

[14] (2019). Clinical Practice Guideline for the Treatment of Depression Across Three Age Cohorts.

[15] Si T, Wang P (2014). When is antidepressant polypharmacy appropriate in the treatment of depression?. Shanghai Arch Psychiatry.

[16] Greenberg PE, Fournier AA, Sisitsky T, Simes M, Berman R, Koenigsberg SH, Kessler RC (2021). The economic burden of adults with major depressive disorder in the United States (2010 and 2018). Pharmacoeconomics.

[17] Greenberg P, Chitnis A, Louie D (2023). The economic burden of adults with major depressive disorder in the United States (2019). Adv Ther.

